# Radiological Society of North America (RSNA) 3D Printing Special Interest Group (SIG) clinical situations for which 3D printing is considered an appropriate representation or extension of data contained in a medical imaging examination: abdominal, hepatobiliary, and gastrointestinal conditions

**DOI:** 10.1186/s41205-020-00065-6

**Published:** 2020-06-08

**Authors:** David H. Ballard, Nicole Wake, Jan Witowski, Frank J. Rybicki, Adnan Sheikh, RSNA Special Interest Group for 3D Printing Abdominal, Hepatobiliary, and Gastrointestinal Conditions Voting Group, David H. Ballard, Adnan M. Sheikh, William J. Weadock, Justin R. Ryan, Jane S. Matsumoto, Carolina Souza, Nicole Wake, Dimitry Levine, Anish Ghodadra, Edward P. Quigley, Andy Christensen, Leonid Chepelev, Waleed Althobaithy, Satheesh Jeyaraj, April Krivaniak, Todd Pietila, Rami Shorti, Lumarie Santiago, Elsa Arribas, Summer Decker, Jayanthi Parthasarathy, Jan Witowski

**Affiliations:** 1 Mallinckrodt Institute of Radiology, Washington University School of Medicine, 510 S. Kingshighway Blvd, Campus Box 8131, St. Louis, MO 63110 USA; 2 Department of Radiology, Montefiore Medical Center, Albert Einstein College of Medicine, Bronx, NY USA; 3 2nd Department of General Surgery, Jagiellonian University Medical College, Kopernika 21, 31-501 Krakow, Poland; 4 Department of Radiology, University of Cincinnati Medical Center, Cincinnati, OH USA; 5 Department of Radiology and The Ottawa Hospital Research Institute, University of Ottawa, Ottawa, ON Canada

## Abstract

**Background:**

Medical 3D printing has demonstrated value in anatomic models for abdominal, hepatobiliary, and gastrointestinal conditions. A writing group composed of the Radiological Society of North America (RSNA) Special Interest Group on 3D Printing (SIG) provides appropriateness criteria for abdominal, hepatobiliary, and gastrointestinal 3D printing indications.

**Methods:**

A literature search was conducted to identify all relevant articles using 3D printing technology associated with a number of abdominal pathologic processes. Each included study was graded according to published guidelines.

**Results:**

Evidence-based appropriateness guidelines are provided for the following areas: intra-hepatic masses, hilar cholangiocarcinoma, biliary stenosis, biliary stones, gallbladder pathology, pancreatic cancer, pancreatitis, splenic disease, gastric pathology, small bowel pathology, colorectal cancer, perianal fistula, visceral trauma, hernia, abdominal sarcoma, abdominal wall masses, and intra-abdominal fluid collections.

**Conclusion:**

This document provides initial appropriate use criteria for medical 3D printing in abdominal, hepatobiliary, and gastrointestinal conditions.

## Background

In 2018, the Radiological Society of North America (RSNA) Special Interest Group on 3D Printing (SIG) published initial guidelines for medical 3D printing appropriateness [1]. Those appropriateness guidelines included a number of organ or system-based appropriateness criteria; however, they did not include indications for abdominal, hepatobiliary, and gastrointestinal 3D printing. Medical 3D printing has been gaining popularity in new areas of clinical practice and is now performed for a variety of abdominal indications [2]. However, there is no consensus on which abdominal, hepatobiliary, and gastrointestinal scenarios and indications can most benefit from 3D printing. The purpose of this work is to provide evidence-based appropriate use criteria for abdominal, hepatobiliary, and gastrointestinal indication for medical 3D printing.

## Methods

The SIG initiated writing groups for appropriateness of performing 3D printing from medical imaging for various clinical conditions. This present work provides the literature search and strength of evidence to introduce the appropriateness of abdominal, hepatobiliary, and gastrointestinal 3D printing for clinical utilization, research, scientific, and informational purposes. Related work previously published and not covered in the present work includes genitourinary and abdominal vascular conditions, which were presented in the initial appropriateness guidelines [1]. This work is loosely modeled after the American College of Radiology (ACR) Appropriateness Criteria® [3], in that the guidelines committee uses an evidence-based approach at scoring. Consensus among members is used when there is a paucity of evidence. Strength of evidence is determined by literature review.

The SIG Guidelines Chairperson oversees the ratings via a vote among Special Interest Group members at in-person meetings. The results of the ratings follow the following 1–9 format (with 9 being the most appropriate):

- 1–3, red, rarely appropriate: There is a lack of a clear benefit or experience that shows an advantage over usual practice.
- 4–6, yellow, may be appropriate: There may be times when there is an advantage, but the data is lacking, or the benefits have not been fully defined.
- 7–9, green, usually appropriate: Data and experience shows an advantage to 3D printing as a method to represent and/or extend the value of data contained in the medical imaging examination.

Clinical scenarios were organized by organ systems. An exhaustive PubMed literature search was performed through October 2018, a strength of evidence analysis was performed, and an appropriate use criteria document was generated. The supporting evidence was obtained through structured PubMed searches, as detailed in the Appendix 1. For each category, from the pool of total results, the number of publications considered “included results” was initially curated by a single author with expertise in 3D printing and abdominal imaging (DHB) then substantiated by consensus of coauthors with expertise in 3D printing. For the present study, only anatomic models were included for evaluation. The following categories were excluded because they were considered outside the project scope: virtual and augmented reality, 3D printed implants, 3D printed instruments and surgical guides, bioprinting, and bioactive printing. Abdominal 3D printing review articles were recorded, but not considered in determining final appropriateness ratings. All final components of this section were vetted and approved by vote of Special Interest Group members face-to-face at the 2018 Annual Meeting of the Radiological Society of North America (November 27, 2018, Chicago, IL, USA). Afterwards, a 2-week period for comments by SIG member was posted on the SIG’s members-only online forum. In addition, all included studies [4–49] were graded with a strength of evidence assessment according to ACR Appropriateness Criteria Evidence Document [50].

## Results

Table 1 provides evidence-based guidelines, supplemented by expert opinion when there was a paucity of peer-review data, to define and support the use of 3D printing for patients with abdominal, hepatobiliary, and gastrointestinal conditions. The citations included in forming the appropriateness criteria and the strength of evidence assessment are presented in Appendices 1 and 2 respectively.

**Table 1:**
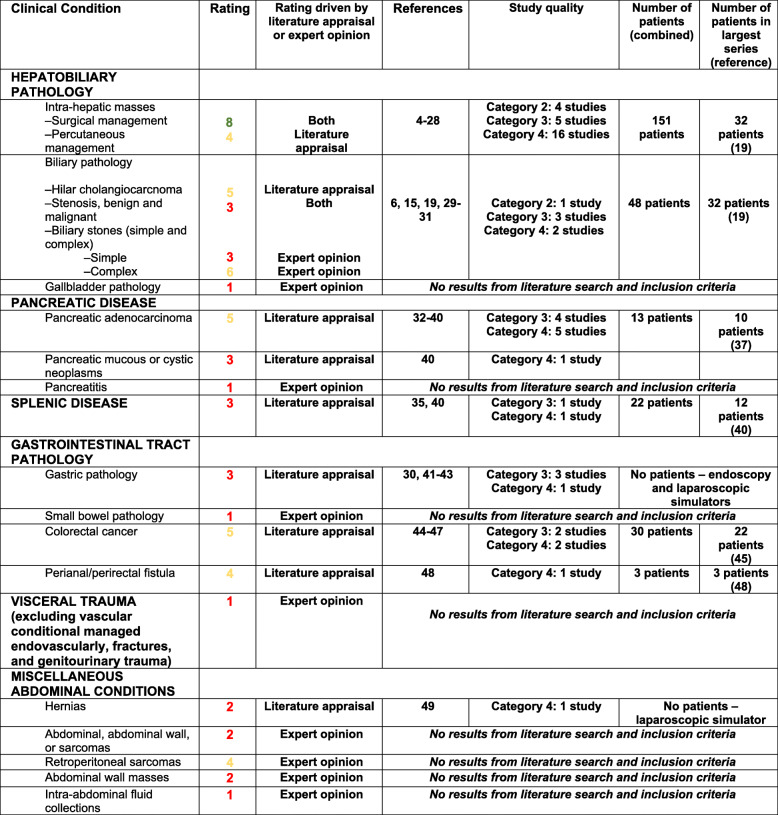
Appropriateness Ratings for Abdominal, Hepatobiliary, and Gastrointestinal Indications. The “Rating driven…” column denotes if the primary decision for the condition’s rating was decided primarily through results and discussion of the literature search or expert opinion (the latter was largely reserved for conditions with no or few supporting studies). The “Study quality” column reflects the graded strength of evidence assessment according to ACR Appropriateness Criteria Evidence Document^50^ (individual ratings available in Appendix 2). The highest/most robust level of evidence is ‘Category 1’ and the lowest is ‘Category 4.’ No studies qualified for Category 1, but multiple did qualify for Category 2

## Discussion

### Hepatobiliary

The majority of intrahepatic masses requiring resection in adults includes hepatocellular carcinoma and isolated or few intrahepatic metastases, such as colorectal metastasis [51]. Anatomic models have been used in preoperative planning for surgical resection of hepatic masses [19, 21, 52]. Specifically, 3D printed anatomic models may be helpful in the resection of hepatic tumors through demonstrating the relationship of the tumor in regards to its location within hepatic segments, invasion or proximity to major hepatic or portal veins, arteries, and bile ducts. Printed models can help in choosing the optimal resection plane and may be useful in selecting patients at risk of posthepatectomy liver failure. Additionally, anatomic models have been used for both liver transplant donor and recipients [20].

Biliary obstruction has benign and malignant etiologies including strictures, extrinsic compression, stones, and biliary malignancies. Symptomatic patients or those with laboratory derangements of obstructive jaundice or liver dysfunction may benefit from endoscopic stones and sludge removal, dilation, and stenting in select cases [53]. For biliary endoscopy, previous work show 3D printed anatomic models being used primarily in training applications [29, 30]. 3D printing has been used in the development of novel biliary stents [54].

Laparoscopic cholecystectomy is among one of the most common operations performed by general surgeons, which may be performed in an acute setting for acute cholecystitis or electively for symptomatic cholelithiasis and other indications [55]. Percutaneous cholecystostomy is a percutaneous approach for management of acute cholecystitis, often performed in those who are critically ill or poor candidates for general anesthesia [56]. The gallbladder may infrequently be a site of primary malignancy, prompting cholecystectomy (often open and radical) in the absence of metastasis. Specific applications of 3D printed anatomic models related to the gallbladder have not been published through the extent of our literature search.

### Pancreas and spleen

Pancreatic cancer is the fourth leading cause of cancer death in the United States and typically caries a poor prognosis with a 3% 5-year survival rate [57]. Resections of pancreatic tumors are challenging operations with high rates of morbidity [58]. To this end, 3D printed anatomic models to delineate tumor anatomy may be of use and have been published in cases series for pancreatic cancer applications [34–37].

There are a number of mucinous and serous pancreatic neoplasms, some of which may be indicated for surgical resection [59]. One educational series fabricated a 3D printed anatomic model for a pancreatic tail mucinous neoplasm, although this was not used in the patient’s preoperative planning [34].

Pancreatitis is an inflammatory response of the pancreas most commonly due to alcoholism and obstruction of the pancreatic duct. This condition is a clinical diagnosis by symptomatology and laboratory derangements. Imaging can be used to help confirm the diagnosis or assess for complications. Complications requiring percutaneous, endoscopic, and infrequently surgical management include infected peripancreatic fluid collections, walled off pancreatic necrosis, and other less frequent etiologies [60]. Specific applications of 3D printed anatomic models related to pancreatitis or its complications have not been published through the extent of our literature search.

Elective splenectomy is often performed for hematologic conditions, as part of larger operations (typically cancer adjacent cancer resections), or, rarely, due to splenic masses with indications for resection. There have been two cases series involving 3D printed anatomic models [36, 40], one of which was used in the process of patient consent [40].

### Gastrointestinal

The incidence of gastric cancer has decreased worldwide with improved detection and treatment of Helicobacter pylori and availability of endoscopy. However, gastric cancer remains a morbid diagnosis and cause of cancer death [61]. Peptic ulcer disease is a prevalent condition, often treated medically and occasionally further characterized with endoscopy [53]. Published uses of anatomic models in gastric pathology largely encompass simulation of endoscopy or surgery [41, 42].

Small bowel tumors are relatively uncommon, most commonly due to adenocarcinoma, gastrointestinal stromal tumor, carcinoid tumor and lymphoma. These may present with abdominal pain, small bowel obstruction, or without symptoms [62]. In North America, small bowel obstruction most commonly occurs due to postoperative adhesions. Other causes include incarcerated hernias, strictures, and malignant obstruction [63]. Specific applications of 3D printed anatomic models related to small bowel pathology have not been published through the extent of the literature search.

Colorectal cancer is the third most common cause of cancer death in the United States [57]. Treatment strategies vary considerably according to anatomic location, staging, among other factors [64]. 3D printed anatomic models have shown some utility in delineating relevant surgical anatomy for resection of colorectal cancer [45, 46].

Anorectal fistulae and abscesses are abnormal tracts and collections about the anus and rectum that occur with greater frequency in patients with Crohn disease [65]. Frequently, pelvic MRI may be obtained to delineate anatomy for treatment planning. In one feasibility series, 3D printed models were used to demonstrate anatomy of anorectal fistulae [48].

### Visceral abdominal trauma

Blunt and penetrating abdominal trauma may result in life-threatening visceral trauma requiring resuscitative efforts and exploratory laparotomy. With current 3D printing technology, the time needed to segment and print anatomic models is currently too lengthy for use in traumatic conditions requiring immediate treatment. Accordingly, our literature search yielded no relevant results regarding the use of 3D printed anatomic models in visceral abdominal trauma.

### Miscellaneous abdominal conditions

Elective hernia repair is among the most common operations performed by general surgeons [66]. Diagnosis is often by physical examination and imaging infrequently plays a part in diagnosing hernias. However, although anatomic models have not been published for preoperative planning of hernias, 3D printing has facilitated a training system in one published series [49]. 3D printing technologies have been used in the design of novel surgical meshes [67, 68].

Sarcomas are aggressive tumors, often locally advanced at the time of diagnosis. They may occur anywhere in the body. In the abdomen, sarcomas may be retroperitoneal, intra-abdominal, or affect the abdominal wall [69]. Although 3D printed anatomic models could potentially be helpful in planning surgical approaches, our search yielded no results for abdominal sarcomas. One case series described anatomic models used for treatment planning in a mediastinal/intrathoracic sarcoma [70].

Abdominal wall masses are most frequently benign and include fibromatosis (desmoid tumor) and endometriosis. Malignant causes are most frequently metastases or sarcomas [69, 71]. Specific applications of 3D printed anatomic models related to abdominal wall masses have not been published through the extent of our literature search.

Intra-abdominal fluid collections with indications for drainage are frequently managed with image-guided percutaneous drainage [72]. There is often acuity in time to drainage, which may account for the lack of publications related to 3D printed anatomic models delineating intra-abdominal fluid collections. Accordingly, our literature search yielded no results.

### Limitations

Limitations of this work include its lack of objective data collection and inferential statistics. Although such an analysis would be desirable, it is not practical with most published abdominal, hepatobiliary, and gastrointestinal applications due to the small number of publications and patients. One exception is 3D printing in liver surgery, which does have a previously published systematic review [73]. PubMed search terms, as highlighted in Appendix 1, were based on prior search terminology from previously published guidelines [1] and used ‘3D printing’ or ‘rapid prototyping’ to capture 3D printing-related publications; it is possible some publications may have been missed without using additional terms such as ‘three dimensional printing’ or ‘three-dimensional printing’. The RSNA 3D Printing SIG is comprised of physicians (primarily radiologists), imaging scientists, biomedical engineers, and other 3D printing experts, the voting group did not have direct input from general surgeons, gastroenterologists, or collaboration from a surgery or gastroenterology professional organization. Future iterations should aim for such collaboration.

## Conclusion

This document provides initial appropriate use criteria for 3D printing in abdominal, hepatobiliary, and gastrointestinal conditions. Adoption of common clinical standards regarding appropriate use, information and material management, and quality control are needed to ensure the greatest possible clinical benefit from 3D printing [1]. With accruing evidence for value in 3D printing, recently implemented category III Current Procedural Terminology codes, and the upcoming ACR registry for 3D printing [74], it is anticipated that this consensus guideline document, created by the members of the RSNA 3D printing Special Interest Group, will provide a reference for clinical standards of 3D printing. The document will be periodically refined, based on expanding clinical applications and growing medical literature.

## Supplementary information

**Additional file 1: Appendix 1.** Literature search

**Additional file 2: Appendix 2.** Strength of Evidence

## Data Availability

The datasets used and/or analyzed during the current study are available from the corresponding author on reasonable request.
