# Supplementary material for: Radiological Society of North America (RSNA) 3D Printing Special Interest Group (SIG) clinical situations for which 3D printing is considered an appropriate representation or extension of data contained in a medical imaging examination: abdominal, hepatobiliary, and gastrointestinal conditions
Source: 3D Print Med. 2020 Jun 8;6:13. doi: 10.1186/s41205-020-00065-6 (PMC7278118; doi:10.1186/s41205-020-00065-6)
Supplement: Supplementary file 1 — Additional file 1: Appendix 1. Literature search [file 41205_2020_65_MOESM1_ESM.docx]

# **Appendix 1: Literature search**

**Hepatobiliary pathology, excluding trauma**

1. **Liver cancer, hepatocellular carcinoma, and hepatomas:**

**Terms:** Either **‘3D printing’** or ‘**rapid prototyping’** (without quotes) individually paired (using Boolean operator AND) with: hepatoma, hepatocellular carcinoma, cholangiocarcinoma, liver cancer, hepatectomy, liver metastasis, intrahepatic metastasis

**PubMed search:** (3D printing AND hepatoma) OR (3D printing AND hepatocellular carcinoma) OR (3D printing AND cholangiocarcinoma) OR (3D printing AND liver cancer) OR (3D printing AND hepatectomy) OR (3D printing AND liver metastasis) OR (3D printing AND intrahepatic metastasis) OR (rapid prototyping AND hepatoma) OR (rapid prototyping AND hepatocellular carcinoma) OR (rapid prototyping AND cholangiocarcinoma) OR (rapid prototyping AND liver cancer) OR (rapid prototyping AND hepatectomy) OR (rapid prototyping AND liver metastasis) OR (rapid prototyping AND intrahepatic metastasis)

**Overall results**: There were 51 overall results. Out of these, 25 were deemed as relevant and included as supporting references. The remaining 26 excluded as they did not use anatomic models.

**Table S1.**

| Article category | Number of article types | Number of patients | Used in clinical care | Notes |
| --- | --- | --- | --- | --- |
| Case reports | 12 | 13 | Yes=8, no=1, unclear=3 |  |
| Case series | 9 | 112 | Yes=5, No=1, Indirectly=2, Unclear=1 | Indirectly = used in patient consent and used for pathologic sectioning |
| Review articles | 4 | NA | No |  |
| Other | 1 | NA | No | Image-guide percutaneous procedural training/phantom study |

**Table S1**. Article categories for hepatobiliary pathology

**List of included relevant hepatobiliary references (n=25):**

1. Igami T, Nakamura Y, Hirose T, et al. Application of a three-dimensional print of a liver in hepatectomy for small tumors invisible by intraoperative ultrasonography: preliminary experience. World J Surg. 2014;38(12):3163–3166.
2. Baimakhanov Z, Soyama A, Takatsuki M, et al. Preoperative simulation with a 3-dimensional printed solid model for one-step reconstruction of multiple hepatic veins during living donor liver transplantation. Liver Transpl. 2015;21(2):266–268.
3. Takagi K, Nanashima A, Abo T, et al. Three-dimensional printing model of liver for operative simulation in perihilar cholangiocarcinoma. Hepatogastroenterology. 2014;61(136):2315–2316.
4. Souzaki R, Kinoshita Y, Ieiri S, et al. Three-dimensional liver model based on preoperative CT images as a tool to assist in surgical planning for hepatoblastoma in a child. Pediatr Surg Int. 2015;31(6):593–596.
5. Xiang N, Fang C, Fan Y, et al. Application of liver three-dimensional printing in hepatectomy for complex massive hepatocarcinoma with rare variations of portal vein: preliminary experience. Int J Clin Exp Med. 2015;8(10):18873–18878.
6. Oshiro Y, Mitani J, Okada T, Ohkohchi N. A novel three-dimensional print of liver vessels and tumors in hepatectomy. Surg Today. 2017;47(4):521–524.
7. Soejima Y, Taguchi T, Sugimoto M, et al. Three-dimensional printing and biotexture modeling for preoperative simulation in living donor liver transplantation for small infants. Liver Transpl. 2016;22(11):1610–1614.
8. Madurska MJ, Poyade M, Eason D, Rea P, Watson AJM. Development of a Patient-Specific 3D-Printed Liver Model for Preoperative Planning. Surg Innov. 2017;24(2):145–150.
9. Perica E, Sun Z. Patient-specific three-dimensional printing for pre-surgical planning in hepatocellular carcinoma treatment. Quant Imaging Med Surg. 2017;7(6):668–677.
10. Kuroda S, Kobayashi T, Ohdan H. 3D printing model of the intrahepatic vessels for navigation during anatomical resection of hepatocellular carcinoma. Int J Surg Case Rep. 2017;41:219–222.
11. Witowski JS, Pędziwiatr M, Major P, Budzyński A. Cost-effective, personalized, 3D-printed liver model for preoperative planning before laparoscopic liver hemihepatectomy for colorectal cancer metastases. Int J Comput Assist Radiol Surg. 2017;12(12):2047–2054.
12. Yang Y, Zhou Z, Liu R, Chen L, Xiang H, Chen N. Application of 3D visualization and 3D printing technology on ERCP for patients with hilar cholangiocarcinoma. Exp Ther Med. 2018;15(4):3259–3264.
13. Choi YR, Kim JH, Park SJ, Hur BY, Han JK. Therapeutic response assessment using 3D ultrasound for hepatic metastasis from colorectal cancer: Application of a personalized, 3D-printed tumor model using CT images. PLoS ONE. 2017;12(8):e0182596.
14. Trout AT, Batie MR, Gupta A, Sheridan RM, Tiao GM, Towbin AJ. 3D printed pathological sectioning boxes to facilitate radiological-pathological correlation in hepatectomy cases. J Clin Pathol. 2017;70(11):984–987.
15. Yang T, Tan T, Yang J, et al. The impact of using three-dimensional printed liver models for patient education. J Int Med Res. 2018;46(4):1570–1578.
16. Zeng N, Fang CH, Fan YF, et al. [The construction of three-dimensional visualization platform and its application in diagnosis and treatment for hilar cholangiocarcinoma]. Zhonghua Wai Ke Za Zhi. 2016;54(9):680–685.
17. Fang C, Fang Z, Fan Y, Li J, Xiang F, Tao H. [Application of 3D visualization, 3D printing and 3D laparoscopy in the diagnosis and surgical treatment of hepatic tumors]. Nan Fang Yi Ke Da Xue Xue Bao. 2015;35(5):639–645.
18. Zein NN, Hanouneh IA, Bishop PD, et al. Three-dimensional print of a liver for preoperative planning in living donor liver transplantation. Liver Transpl. 2013;19(12):1304–1310.
19. Hu M, Hu H, Cai W, et al. The Safety and Feasibility of Three-Dimensional Visualization Technology Assisted Right Posterior Lobe Allied with Part of V and VIII Sectionectomy for Right Hepatic Malignancy Therapy. J Laparoendosc Adv Surg Tech A. 2018;28(5):586–594.
20. Wang J-Z, Xiong N-Y, Zhao L-Z, Hu J-T, Kong D-C, Yuan J-Y. Review fantastic medical implications of 3D-printing in liver surgeries, liver regeneration, liver transplantation and drug hepatotoxicity testing: A review. Int J Surg. 2018;56:1–6.
21. Oshiro Y, Ohkohchi N. Three-Dimensional Liver Surgery Simulation: Computer-Assisted Surgical Planning with Three-Dimensional Simulation Software and Three-Dimensional Printing<sup/>. Tissue Eng Part A. 2017;23(11–12):474–480.
22. Yao R, Xu G, Mao S-S, et al. Three-dimensional printing: review of application in medicine and hepatic surgery. Cancer Biol Med. 2016;13(4):443–451.
23. Alkhouri N, Zein NN. Three-dimensional printing and pediatric liver disease. Curr Opin Pediatr. 2016;28(5):626–630.
24. Widmann G, Wallach D, Toporek G, Schullian P, Weber S, Bale R. Angiographic C-arm CT- versus MDCT-guided stereotactic punctures of liver lesions: nonrigid phantom study. AJR Am J Roentgenol. 2013;201(5):1136–1140.
25. Witowski J, Wake N, Grochowska A, et al. Investigating accuracy of 3D printed liver models with computed tomography. Quant Imaging Med Surg. 2019;9(1):43-52.
26. **Biliary stenosis (benign and malignant) and biliary stones, endoscopic and percutaneous management:**

**Terms:** Either **‘3D printing’** or ‘**rapid prototyping’** (without quotes) individually paired (using Boolean operator AND) with: bile ducts, biliary stenosis, bile duct stenosis, bile duct stenting, biliary stenting, endoscopic retrograde cholangiopancreatography, ERCP, cholangiocarcinoma, hilar cholangiocarcinoma, common bile duct, choledocholithiasis, biliary endoscopy

**PubMed search**: (3D printing AND bile ducts) OR (3D printing AND biliary stenosis) OR (3D printing AND bile duct stenosis) OR (3D printing AND bile duct stenting) OR (3D printing AND biliary stenting) OR (3D printing AND endoscopic retrograde cholangiopancreatography) OR (3D printing AND ERCP) OR (3D printing AND cholangiocarcinoma) OR (3D printing AND hilar cholangiocarcinoma) OR (3D printing AND common bile duct) OR (3D printing AND choledocholithiasis) OR (3D printing AND biliary endoscopy) OR (rapid prototyping AND bile ducts) OR (rapid prototyping AND biliary stenosis) OR (rapid prototyping AND bile duct stenosis) OR (rapid prototyping AND bile duct stenting) OR (rapid prototyping AND biliary stenting) OR (rapid prototyping AND endoscopic retrograde cholangiopancreatography) OR (rapid prototyping AND ERCP) OR (rapid prototyping AND cholangiocarcinoma) OR (rapid prototyping AND hilar cholangiocarcinoma) OR (rapid prototyping AND common bile duct) OR (rapid prototyping AND choledocholithiasis) OR (rapid prototyping AND biliary endoscopy)

**Overall results**: 10 results with 4 included as supporting references and 6 excluded that did not use anatomic models.

**Table S2.**

| Article category | Number of article types | Number of patients | Used in clinical care | Notes |
| --- | --- | --- | --- | --- |
| Training/simulation | 3 | NA | No |  |
| Case series | 1 | 15 | Unclear | Hilar cholangicarcinoma |

**Table S2**. Articles for biliary pathology

**List of included relevant biliary references (n=4):**

- 1. Tang R, Ma L, Li A, et al. Choledochoscopic Examination of a 3-Dimensional Printing Model Using Augmented Reality Techniques: A Preliminary Proof of Concept Study. Surg Innov. 2018;25(5):492–498.
  2. Yang Y, Zhou Z, Liu R, Chen L, Xiang H, Chen N. Application of 3D visualization and 3D printing technology on ERCP for patients with hilar cholangiocarcinoma. Exp Ther Med. 2018;15(4):3259–3264.
  3. Holt BA, Hearn G, Hawes R, Tharian B, Varadarajulu S. Development and evaluation of a 3D printed endoscopic ampullectomy training model (with video). Gastrointest Endosc. 2015;81(6):1470-1475.e5.
  4. Dhir V, Itoi T, Fockens P, et al. Novel ex vivo model for hands-on teaching of and training in EUS-guided biliary drainage: creation of “Mumbai EUS” stereolithography/3D printing bile duct prototype (with videos). Gastrointest Endosc. 2015;81(2):440–446.

1. **Gallbladder pathology, surgical or percutaneous management:**

**Terms:** Either **‘3D printing’** or ‘**rapid prototyping’** (without quotes) individually paired (using Boolean operator AND) with: gallbladder, cholecystectomy, laparoscopic cholecystectomy, open cholecystectomy, cholelithiasis, gallstones, biliary colic, gallbladder sludge, biliary sludge, gallbladder cancer, gallbladder adenocarcinoma, cholecystostomy, percutaneous cholecystostomy)

**PubMed search**: (3D printing AND gallbladder) OR (3D printing AND cholecystectomy) OR (3D printing AND laparoscopic cholecystectomy) OR (3D printing AND open cholecystectomy) OR (3D printing AND cholelithiasis) OR (3D printing AND gallstones) OR (3D printing AND biliary colic) OR (3D printing AND gallbladder sludge) OR (3D printing AND biliary sludge) OR (3D printing AND gallbladder cancer) OR (3D printing AND gallbladder adenocarcinoma) OR (3D printing AND cholecystostomy) OR (3D printing AND percutaneous cholecystostomy) OR (rapid prototyping AND gallbladder) OR (rapid prototyping AND cholecystectomy) OR (rapid prototyping AND laparoscopic cholecystectomy) OR (rapid prototyping AND open cholecystectomy) OR (rapid prototyping AND cholelithiasis) OR (rapid prototyping printing AND gallstones) OR (rapid prototyping AND biliary colic) OR (rapid prototyping AND gallbladder sludge) OR (rapid prototyping AND biliary sludge) OR (rapid prototyping AND gallbladder cancer) OR (rapid prototyping AND gallbladder adenocarcinoma) OR (rapid prototyping AND cholecystostomy) OR (rapid prototyping AND percutaneous cholecystostomy)

**Overall results**: 19 results were yielded, none were included as relevant.

**There were no relevant search results for anatomic models used or potentially used in patient care.**

**Pancreatic pathology, excluding trauma**

1. **Pancreatic cancer, pancreatic mucinous or serous neoplasms and pancreatitis, acute or chronic, and associated complications (excluding pseudoaneurysms managed endovascularly):**

**Terms:** Either **‘3D printing’** or ‘**rapid prototyping’** (without quotes) individually paired (using Boolean operator AND) with: pancreas, pancreatic adenocarcinoma, pancreatic cancer, pancreatic neuroendocrine tumor, Whipple, Whipple procedure, pancreaticoduodenectomy, distal pancreatectomy, pancreatic surgery, pancreatectomy, intraductal papillary mucinous neoplasms, IPMN, serous cystic adenoma, mucinous cystic neoplasm, solid pseudopapillary epithelial neoplasm, solid pseudopapillary neoplasia, pancreatitis, acute pancreatitis, chronic pancreatitis, pseudocyst, pancreatic pseudocyst, walled-off pancreatic necrosis, peripancreatic fluid collection, pancreatic abscess, pancreatic fistula, pancreatic duct leak pancreatic drainage, necrosectomy, pancreatic necrosectomy)

**PubMed search**: (3D printing AND pancreas) OR (3D printing AND pancreatic adenocarcinoma) OR (3D printing AND pancreatic cancer) OR (3D printing AND pancreatic neuroendocrine tumor) OR (3D printing AND Whipple) OR (3D printing AND Whipple procedure) OR (3D printing AND pancreaticoduodenectomy) OR (3D printing AND distal pancreatectomy) OR (3D printing AND pancreatic surgery) OR (3D printing AND pancreatectomy) OR (3D printing AND intraductal papillary mucinous neoplasms) OR (3D printing AND IPMN) OR (3D printing AND serous cystic adenoma) OR (3D printing AND mucinous cystic neoplasm) OR (3D printing AND solid pseudopapillary epithelial neoplasm) OR (3D printing AND solid pseudopapillary neoplasia) OR (3D printing AND pancreatitis) OR (3D printing AND acute pancreatitis) OR (3D printing AND chronic pancreatitis) OR (3D printing AND pseudocyst) OR (3D printing AND pancreatic pseudocyst) OR (3D printing AND walled-off pancreatic necrosis) OR (3D printing AND peripancreatic fluid collection) OR (3D printing AND pancreatic abscess) OR (3D printing AND pancreatic fistula) OR (3D printing AND pancreatic duct leak) OR (3D printing AND pancreatic drainage) OR (3D printing AND necrosectomy) OR (3D printing AND pancreatic necrosectomy) OR (rapid prototyping AND pancreas) OR (rapid prototyping AND pancreatic adenocarcinoma) OR (rapid prototyping AND pancreatic cancer) OR (rapid prototyping AND pancreatic neuroendocrine tumor) OR (rapid prototyping AND Whipple) OR (rapid prototyping AND Whipple procedure) OR (rapid prototyping AND pancreaticoduodenectomy) OR (rapid prototyping AND distal pancreatectomy) OR (rapid prototyping AND pancreatic surgery) OR (rapid prototyping AND pancreatectomy) OR (rapid prototyping AND intraductal papillary mucinous neoplasms) OR (rapid prototyping AND IPMN) OR (rapid prototyping AND serous cystic adenoma) OR (rapid prototyping AND mucinous cystic neoplasm) OR (rapid prototyping AND solid pseudopapillary epithelial neoplasm) OR (rapid prototyping AND solid pseudopapillary neoplasia) OR (rapid prototyping AND pancreatitis) OR (rapid prototyping AND acute pancreatitis) OR (rapid prototyping AND chronic pancreatitis) OR (rapid prototyping AND pseudocyst) OR (rapid prototyping AND pancreatic pseudocyst) OR (rapid prototyping AND walled-off pancreatic necrosis) OR (rapid prototyping AND peripancreatic fluid collection) OR (rapid prototyping AND pancreatic abscess) OR (rapid prototyping AND pancreatic fistula) OR (rapid prototyping AND pancreatic duct leak) OR (rapid prototyping AND pancreatic drainage) OR (rapid prototyping AND necrosectomy) OR (rapid prototyping AND pancreatic necrosectomy)

**Overall results**: 35 results were yielded, 8 were included. Seven of the 8 results were relevant to pancreatic cancer, 1 of 8 was relevant to mucinous or cystic pancreatic neoplasms, and no results were relevant to pancreatitis.

**Table S3.**

| Article category | Number of article types | Number of patients | Used in clinical care | Notes |
| --- | --- | --- | --- | --- |
| Pancreatic cancer | | | | |
| Case series | 3 | 13 | No=3 |  |
| Review articles | 2 | NA | No |  |
| Case report | 1 | 1 | Yes |  |
| Training/educational | 1 | NA | No |  |
|  |  |  |  |  |
| Mucinous or cystic pancreatic neoplasms | | | | |
| Training/educational | 1 | NA | No |  |
| Pancreatitis | | | | |
| No relevant articles from search query. | | | | |

**Table S3.** Articles for pancreatic pathology

**List of included relevant pancreatic references (n=8):**

- 1. Study Group of Pancreatic Surgery in Chinese Society of Surgery of Chinese Medical Association, Pancreatic Committee of Chinese Research Hospital Association, Digital Medicine Branch of Chinese Medical Association, Digital Medicine Committee of Chinese Research Hospital Association. [Expert consensus of precise diagnosis and treatment for pancreatic head cancer using three-dimensional visualization technology]. Zhonghua Wai Ke Za Zhi. 2017;55(12):881–886.
  2. Yang YY, Huang HG. [Development status of three-dimensional printing technology in pancreatic surgery]. Zhonghua Wai Ke Za Zhi. 2017;55(10):795–797.
  3. Marconi S, Pugliese L, Botti M, et al. Value of 3D printing for the comprehension of surgical anatomy. Surg Endosc. 2017;31(10):4102–4110.
  4. Sampogna G, Pugliese R, Elli M, Vanzulli A, Forgione A. Routine clinical application of virtual reality in abdominal surgery. Minim Invasive Ther Allied Technol. 2017;26(3):135–143.
  5. Andolfi C, Plana A, Kania P, Banerjee PP, Small S. Usefulness of Three-Dimensional Modeling in Surgical Planning, Resident Training, and Patient Education. J Laparoendosc Adv Surg Tech A. 2017;27(5):512–515.
  6. Marconi S, Pugliese L, Del Chiaro M, Pozzi Mucelli R, Auricchio F, Pietrabissa A. An innovative strategy for the identification and 3D reconstruction of pancreatic cancer from CT images. Updates Surg. 2016;68(3):273–278.
  7. Zheng Y, Yu D, Zhao J, Wu Y, Zheng B. 3D Printout Models vs. 3D-Rendered Images: Which Is Better for Preoperative Planning? J Surg Educ. 2016;73(3):518–523.
  8. Mahmoud A, Bennett M. Introducing 3-Dimensional Printing of a Human Anatomic Pathology Specimen: Potential Benefits for Undergraduate and Postgraduate Education and Anatomic Pathology Practice. Arch Pathol Lab Med. 2015;139(8):1048–1051.

**Splenic pathology, excluding trauma and pseudoaneurysms managed endovascularly**

1. **Splenic pathology:**

**Terms:** Either **‘3D printing’** or ‘**rapid prototyping’** (without quotes) individually paired (using Boolean operator AND) with: spleen, splenic, splenomegaly, splenosis, splenic lesion, splenic mass, splenectomy

**PubMed search**: (3D printing AND spleen) OR (3D printing AND splenic) OR (3D printing AND splenomegaly) OR (3D printing AND splenosis) OR (3D printing AND splenic lesion) OR (3D printing AND splenic mass) OR (3D printing AND splenectomy) OR (rapid prototyping AND spleen) OR (rapid prototyping AND splenic) OR (rapid prototyping AND splenomegaly) OR (rapid prototyping AND splenosis) OR (rapid prototyping AND splenic lesion) OR (rapid prototyping AND splenic mass) OR (rapid prototyping AND splenectomy)

**Overall results**: The PubMed search yielded 14 results, 2 of which were included as relevant and 12 of which were excluded.

**Table 4.**

| Article category | Number of article types | Number of patients | Used in clinical care | Notes |
| --- | --- | --- | --- | --- |
| Case series | 2 | 22 | No=1, Indirectly=1 | Indirectly= used in patient consent |

**Table 4.** Articles for splenic pathology

**List of included relevant splenic references (n=2):**

1. Marconi S, Pugliese L, Botti M, et al. Value of 3D printing for the comprehension of surgical anatomy. Surg Endosc. 2017;31(10):4102–4110.
2. Pietrabissa A, Marconi S, Peri A, et al. From CT scanning to 3-D printing technology for the preoperative planning in laparoscopic splenectomy. Surg Endosc. 2016;30(1):366–371.

**Gastrointestinal tract pathology**

1. **Gastric pathology:**

**Terms:** Either **‘3D printing’** or ‘**rapid prototyping’** (without quotes) individually paired (using Boolean operator AND) with: stomach, gastric, gastric adenocarcinoma, peptic ulcer disease, gastroparesis, gastric tumor

**PubMed search**: (3D printing AND stomach) OR (3D printing AND gastric) OR (3D printing AND gastric adenocarcinoma) OR (3D printing AND peptic ulcer disease) OR (3D printing AND gastroparesis) OR (3D printing AND gastric tumor) OR (rapid prototyping AND stomach) OR (rapid prototyping AND gastric) OR (rapid prototyping AND gastric adenocarcinoma) OR (rapid prototyping AND peptic ulcer disease) OR (rapid prototyping printing AND gastroparesis) OR (rapid prototyping AND gastric tumor)

**Overall results**: The PubMed search yielded 23 results, 4 of which were included. All 4 studies were either training simulators or phantom studies

**Table S5.**

| Article category | Number of article types | Number of patients | Used in clinical care | Notes |
| --- | --- | --- | --- | --- |
| Training/educational | 3 | NA | No | 74 total participants in 3 studies |
| Other | 1 | NA | No | Phantom study |

**Table S5.** Articles for gastric pathology

**List of included relevant gastric references (n=4):**

1. Williams A, McWilliam M, Ahlin J, Davidson J, Quantz MA, Bütter A. A simulated training model for laparoscopic pyloromyotomy: Is 3D printing the way of the future? J Pediatr Surg. 2018;53(5):937–941.
2. Lee S, Ahn JY, Han M, et al. Efficacy of a Three-Dimensional-Printed Training Simulator for Endoscopic Biopsy in the Stomach. Gut Liver. 2018;12(2):149–157.
3. Holt BA, Hearn G, Hawes R, Tharian B, Varadarajulu S. Development and evaluation of a 3D printed endoscopic ampullectomy training model (with video). Gastrointest Endosc. 2015;81(6):1470-1475.e5.
4. Kim GB, Park J-H, Song H-Y, et al. 3D-printed phantom study for investigating stent abutment during gastroduodenal stent placement for gastric outlet obstruction. 3D Print Med. 2017;3(1):10.
5. **Small bowel pathology:**

**Terms:** Either **‘3D printing’** or ‘**rapid prototyping’** (without quotes) individually paired (using Boolean operator AND) with: small bowel, bowel, small bowel tumor, small bowel stricture, small bowel obstruction

**PubMed search**: (3D printing AND small bowel) OR (3D printing AND bowel) OR (3D printing AND small bowel tumor) OR (3D printing AND small bowel stricture) OR (3D printing AND small bowel obstruction) OR (rapid prototyping AND small bowel) OR (rapid prototyping AND bowel) OR (rapid prototyping AND small bowel tumor) OR (rapid prototyping AND small bowel stricture) OR (rapid prototyping AND small bowel obstruction)

**Overall results**: The PubMed search yielded 17 results, none of which were relevant to anatomic models.

**There were no relevant search results for anatomic models used or potentially used in patient care.**

1. **Colonic, rectal, and anal pathology:**

**Terms:** Either **‘3D printing’** or ‘**rapid prototyping’** (without quotes) individually paired (using Boolean operator AND) with: colon, colonic, rectum, rectal, anus, anal, colon cancer, rectal cancer, colonic obstruction, perirectal abscess, perirectal fistula, perianal abscess, perianal fistula

**PubMed search**: (3D printing AND colon) OR (3D printing AND colonic) OR (3D printing AND rectum) OR (3D printing AND rectal) OR (3D printing AND anus) OR (3D printing AND anal) OR (3D printing AND colon cancer) OR (3D printing AND rectal cancer) OR (3D printing AND colonic obstruction) OR (3D printing AND perirectal abscess) OR (3D printing AND perirectal fistula) OR (3D printing AND perianal abscess) OR (3D printing AND perianal fistula) OR (3D printing AND colectomy) OR (3D printing AND hemicolectomy) OR (3D printing AND colonoscopy) OR (rapid prototyping AND colon) OR (rapid prototyping AND colonic) OR (rapid prototyping AND rectum) OR (rapid prototyping AND rectal) OR (rapid prototyping AND anus) OR (rapid prototyping AND anal) OR (rapid prototyping AND colon cancer) OR (rapid prototyping AND rectal cancer) OR (rapid prototyping AND colonic obstruction) OR (rapid prototyping perirectal abscess) OR (rapid prototyping AND perirectal fistula) OR (rapid prototyping AND perianal abscess) OR (rapid prototyping AND perianal fistula) OR (rapid prototyping AND colectomy) OR (rapid prototyping AND hemicolectomy) OR (rapid prototyping AND colonoscopy)

**Overall results:** The PubMed search yielded 153 results, 5 of which were relevant to anatomic models.

**Table S6.**

| Article category | Number of article types | Number of patients | Used in clinical care | Notes |
| --- | --- | --- | --- | --- |
| Colorectal cancer | | | | |
| Case series | 1 | 22 | No |  |
| Case report | 2 | 2 | Yes=1, No=1 |  |
| Other | 1 | 4 | Indirectly | Ostomy stoma models used to instruct patients on stoma care |
| Perianal/perirectal fistula | | | | |
| Case series | 1 | 3 | No |  |
| Perianal/perirectal abscess | | | | |
| No relevant articles from search query. | | | | |

**Table S6.** Articles for colon and anorectal pathology

**List of included relevant colon and anorectal references (n=5):**

1. Sahnan K, Adegbola SO, Tozer PJ, et al. Innovation in the imaging perianal fistula: a step towards personalised medicine. Therap Adv Gastroenterol. 2018;11:1756284818775060.
2. Garcia-Granero A, Sánchez-Guillén L, Fletcher-Sanfeliu D, et al. Application of three-dimensional printing in laparoscopic dissection to facilitate D3-lymphadenectomy for right colon cancer. Tech Coloproctol. 2018;22(2):129–133.
3. Luzon JA, Andersen BT, Stimec BV, et al. Implementation of 3D printed superior mesenteric vascular models for surgical planning and/or navigation in right colectomy with extended D3 mesenterectomy: comparison of virtual and physical models to the anatomy found at surgery. Surg Endosc. 2018;
4. Hamabe A, Ito M. A three-dimensional pelvic model made with a three-dimensional printer: applications for laparoscopic surgery to treat rectal cancer. Tech Coloproctol. 2017;21(5):383–387.
5. Tominaga T, Takagi K, Takeshita H, et al. Usefulness Of Three-Dimensional Printing Models for Patients with Stoma Construction. Case Rep Gastroenterol. 2016;10(1):57–62.

**Traumatic conditions in the abdomen, excluding fractures and vascular trauma managed endovascularly**

1. **Visceral abdominal trauma, excluding genitourinary trauma:**

**Terms:** Either **‘3D printing’** or ‘**rapid prototyping’** (without quotes) individually paired (using Boolean operator AND) with: abdominal trauma, blunt trauma, penetrating trauma, liver laceration, splenic laceration, small bowel injury, mesenteric injury, traumatic abdominal wall hernia, hemoperitoneum, bowel injury, colonic injury, motor vehicle collision, gunshot wound. Note – ‘trauma’ was not included, see rationale in ‘Overall results.’

**PubMed search**: (3D printing AND abdominal trauma) OR (3D printing AND blunt trauma) OR (3D printing AND penetrating trauma) (3D printing AND liver laceration) OR (3D printing AND splenic laceration) OR (3D printing AND small bowel injury) OR (3D printing AND mesenteric injury) OR (3D printing AND traumatic abdominal wall hernia) OR (3D printing AND hemoperitoneum) OR (3D printing AND bowel injury) OR (3D printing AND colonic injury) OR (3D printing AND motor vehicle collision) OR (3D printing AND gunshot wound) OR (rapid prototyping AND abdominal trauma) OR (rapid prototyping AND blunt trauma) OR (rapid prototyping AND penetrating trauma) OR (3D printing and diaphragm injury) OR (rapid prototyping AND liver laceration) OR (rapid prototyping AND splenic laceration) OR (rapid prototyping AND small bowel injury) OR (rapid prototyping AND mesenteric injury) OR (rapid prototyping AND traumatic abdominal wall hernia) OR (rapid prototyping AND hemoperitoneum) OR (rapid prototyping AND bowel injury) OR (rapid prototyping AND colonic injury) OR (rapid prototyping AND motor vehicle collision) OR (rapid prototyping AND gunshot wound) OR (rapid prototyping and diaphragm injury)

**Overall results**: 19 results were yielded, none were relevant. Note - both of the following search terms were omitted: (3D printing and trauma) OR (rapid prototyping and trauma). Inclusion of these two search terms increased the yield of the above search from 19 results to 550 results.

**There were no relevant search results for anatomic models used or potentially used in patient care.**

**Abdominal conditions, miscellaneous**

1. **Hernias, including ventral or incisional and inguinal:**

**Terms:** Either **‘3D printing’** or ‘**rapid prototyping’** (without quotes) individually paired (using Boolean operator AND) with: hernia, inguinal hernia, ventral hernia, incisional hernia, lumbar hernia, abdominal wall reconstruction

**PubMed search**: (3D printing AND hernia) OR (3D printing AND inguinal hernia) OR (3D printing AND inguinal hernia repair) OR (3D printing AND ventral hernia) (3D printing AND incisional hernia) OR (3D printing AND lumbar hernia) OR (3D printing AND abdominal wall reconstruction) OR (3D printing AND transabdominal preperitoneal inguinal hernia repair) OR (rapid prototyping AND hernia) OR (rapid prototyping AND inguinal hernia) OR (rapid prototyping AND inguinal hernia repair) OR (rapid prototyping AND ventral hernia) (rapid prototyping AND incisional hernia) OR (rapid prototyping AND lumbar hernia) OR (rapid prototyping AND abdominal wall reconstruction) OR (rapid prototyping AND transabdominal preperitoneal inguinal hernia repair)

**Overall results**: The search yielded 4 results, 1 of which was a surgical training simulator for transabdominal preperitoneal inguinal hernia repair.

**Table S7.**

| Article category | Number of article types | Number of patients | Used in clinical care | Notes |
| --- | --- | --- | --- | --- |
| Training/educational | 1 | NA | No | transabdominal preperitoneal inguinal hernia repair simulator; 15 participants in training exercise |

**Table S7.** Articles relevant to hernias

**List of included of relevant hernia reference (n=1):**

1. Nishihara Y, Isobe Y, Kitagawa Y. Validation of newly developed physical laparoscopy simulator in transabdominal preperitoneal (TAPP) inguinal hernia repair. Surg Endosc. 2017;31(12):5429–5435.
2. Abdominal wall, intra-abdominal, and retroperitoneal sarcomas:

**Terms:** Either **‘3D printing’** or ‘**rapid prototyping’** (without quotes) individually paired (using Boolean operator AND) with: sarcoma, abdominal wall sarcoma, abdominal sarcoma, retroperitoneal sarcoma

**PubMed search**: (3D printing AND sarcoma) OR (3D printing AND abdominal wall sarcoma) (3D printing AND abdominal sarcoma) OR (3D printing AND retroperitoneal sarcoma) OR (rapid prototyping AND sarcoma) OR (rapid prototyping AND abdominal wall sarcoma) (rapid prototyping AND abdominal sarcoma) OR (rapid prototyping AND retroperitoneal sarcoma)

**Overall results**: The search yielded 1 result that was not directly relevant to abdominal wall, abdominal, or retroperitoneal sarcomas. The single result was a case series of 3 pediatric patients, including 1 patient with an intra-thoracic/mediastinal sarcoma.^70^

**There were no relevant search results for anatomic models used or potentially used in patient care.**

1. **Abdominal wall masses:**

**Terms:** Either **‘3D printing’** or ‘**rapid prototyping’** (without quotes) individually paired (using Boolean operator AND) with: abdominal wall mass, abdominal wall tumor, fibromatosis, desmoid tumor, abdominal wall endometriosis, abdominal wall metastases

**PubMed search**: (3D printing AND abdominal wall mass) OR (3D printing AND abdominal wall tumor) OR (3D printing AND fibromatosis) OR (3D printing AND desmoid tumor) OR (3D printing AND abdominal wall endometriosis) OR (rapid prototyping AND abdominal wall metastases) OR (rapid prototyping AND abdominal wall mass) OR (rapid prototyping AND abdominal wall tumor) OR (rapid prototyping printing AND fibromatosis) OR (rapid prototyping AND desmoid tumor) OR (rapid prototyping AND abdominal wall endometriosis) OR (rapid prototyping AND abdominal wall metastases)

**Overall results**: The search yielded 8 results, none of which were relevant to abdominal wall masses and 3D printing.

**There were no relevant search results for anatomic models used or potentially used in patient care.**

1. **Intra-abdominal fluid collections, percutaneous and surgical management:**

**Terms:** Either **‘3D printing’** or ‘**rapid prototyping’** (without quotes) individually paired (using Boolean operator AND) with: abdominal abscess, intra-abdominal abscess, percutaneous drainage, abdominal fluid collection, intra-abdominal fluid collection

**PubMed search**: (3D printing AND abdominal abscess) OR (3D printing AND intra-abdominal abscess) OR (3D printing AND percutaneous drainage) OR (3D printing AND abdominal fluid collection) OR (3D printing AND intra-abdominal fluid collection) OR (rapid prototyping AND abdominal abscess) OR (rapid prototyping AND intra-abdominal abscess) OR (rapid prototyping AND percutaneous drainage) OR (rapid prototyping printing AND abdominal fluid collection) OR (rapid prototyping AND intra-abdominal fluid collection**)**

**Overall results**: The search yielded 2 results, neither of which were relevant to intra-abdominal fluid collections and 3D printed anatomic models. Of note, one excluded result was a 3D printed abdominal compression device to facilitate percutaneous procedures - Epelboym Y, Shyn PB, Hosny A, et al. Use of a 3D-Printed Abdominal Compression Device to Facilitate CT Fluoroscopy-Guided Percutaneous Interventions. AJR Am J Roentgenol. 2017;209(2):435–441.

**There were no relevant search results for anatomic models used or potentially used in patient care.**
