# Supplementary material for: Radiological Society of North America (RSNA) 3D Printing Special Interest Group (SIG) clinical situations for which 3D printing is considered an appropriate representation or extension of data contained in a medical imaging examination: abdominal, hepatobiliary, and gastrointestinal conditions
Source: 3D Print Med. 2020 Jun 8;6:13. doi: 10.1186/s41205-020-00065-6 (PMC7278118; doi:10.1186/s41205-020-00065-6)
Supplement: Supplementary file 2 — Additional file 2: Appendix 2. Strength of Evidence [file 41205_2020_65_MOESM2_ESM.docx]

**Appendix 2: Strength of Evidence**

| **Reference** | | **Study Type** | **Patients/**  **Events** | **Study Objective**  **(Purpose of Study)** | **Study Results** | **Study Quality** |
| --- | --- | --- | --- | --- | --- | --- |
| 4 | Igami T, Nakamura Y, Hirose T, et al. Application of a three-dimensional print of a liver in hepatectomy for small tumors invisible by intraoperative ultrasonography: preliminary experience. World J Surg. 2014;38(12):3163–3166. | Review/Other-Tx | 2 | 3D printed liver model was used to perform a hepatectomy on two patients with synchronous multiple liver metastases from colorectal cancer, invisible to intraoperative ultrasonography. | Hepatectomy for the small tumor that is invisible to intraoperative ultrasonography was performed by referring to a 3D-printed model. The planned resections were successful with histologically negative surgical margins. Authors claimed that 3D printed models made procedure “easy and feasible” | 4 |
| 5 | Baimakhanov Z, Soyama A, Takatsuki M, et al. Preoperative simulation with a 3-dimensional printed solid model for one-step reconstruction of multiple hepatic veins during living donor liver transplantation. Liver Transpl. 2015;21(2):266–268. | Review/Other-Dx | 1 | Preoperative 3‐dimensional (3D) images were obtained with a 3D image analysis system so that we could evaluate the graft volume and possible congested volume after implantation in LDLT. | Printed model helped to understand spatial representation of complex LDLT anastomosis and choose optimal surgical strategy. | 4 |
| 6 | Takagi K, Nanashima A, Abo T, et al. Three-dimensional printing model of liver for operative simulation in perihilar cholangiocarcinoma. Hepatogastroenterology. 2014;61(136):2315–2316. | Review/Other-Tx | 1 | 3-dimensional printed liver was constructed using 3D vascular imaging in a patient with intrahepatic cholangiocarcinoma who underwent major hepatectomy. | No results stated in abstract. | 4 |
| 7 | Souzaki R, Kinoshita Y, Ieiri S, et al. Three-dimensional liver model based on preoperative CT images as a tool to assist in surgical planning for hepatoblastoma in a child. Pediatr Surg Int. 2015;31(6):593–596. | Review/Other-Tx | 1 | 3D printed liver model was used to determine resectability and simulate surgical procedure in 3-year-old female with hepatoblastoma and atrophied left portal vein.. | Authors stated that 3D printed liver model helped to assess tumor resectability and patient’s abnormal hepatic anatomy before performing successful extended left lobectomy. | 4 |
| 8 | Xiang N, Fang C, Fan Y, et al. Application of liver three-dimensional printing in hepatectomy for complex massive hepatocarcinoma with rare variations of portal vein: preliminary experience. Int J Clin Exp Med. 2015;8(10):18873–18878. | Review/Other-Tx | 1 | To discuss the role of liver 3D printing in the treatment of complex massive hepatocarcinoma with rare variations of portal vein. | 3D printed liver model was consistent with virtual reconstruction of the anatomy. Model aided surgical planning and was also used during the procedure. | 4 |
| 9 | Oshiro Y, Mitani J, Okada T, Ohkohchi N. A novel three-dimensional print of liver vessels and tumors in hepatectomy. Surg Today. 2017;47(4):521–524. | Review/Other-Dx and Tx | NA | To develop 3D low-cost printed liver model with visible anatomy relationships. | A method to develop low-cost liver models was presented. Estimated cost of single model is $600. Hepatectomy was performed using 3D printed model and it helped to simulate the resection line and perform surgery safely. | 4 |
| 10 | Soejima Y, Taguchi T, Sugimoto M, et al. Three-dimensional printing and biotexture modeling for preoperative simulation in living donor liver transplantation for small infants. Liver Transpl. 2016;22(11):1610–1614. | Review/Other-Tx | 1 | Authors describe their experience of pediatric LDLT for a small infant using biotexture models of a liver graft and the recipient's body that were created by a 3D printer. | Prototype of left lateral segment graft was developed and used in planning LDLT. | 4 |
| 11 | Madurska MJ, Poyade M, Eason D, Rea P, Watson AJM. Development of a Patient-Specific 3D-Printed Liver Model for Preoperative Planning. Surg Innov. 2017;24(2):145–150. | Review/Other-Tx | 1 | This article describes the development of models of hepatic structures specific to a patient diagnosed with an operable hepatic malignancy. | The final models of the liver structures and tumor provided good anatomical detail and representation of the spatial relationships between the liver tumor and adjacent hepatic structures and could be easily manipulated and explored from different angles. | 4 |
| 12 | Perica E, Sun Z. Patient-specific three-dimensional printing for pre-surgical planning in hepatocellular carcinoma treatment. Quant Imaging Med Surg. 2017;7(6):668–677. | Review/Other-Tx | 1 | The aim of this study is to investigate the feasibility of utilising 3D printed liver models as clinical tools in pre-operative planning for resectable hepatocellular carcinoma (HCC) lesions. | A scaled down multi-material 3D liver model delineating patient-specific hepatic anatomy and pathology was produced, requiring a total production time of 25.25 hours and costing a total of AUD $1,250. A discrepancy was found in the total mean of measurements at each stage of production, with a total mean of 18.28±9.31 mm for measurements acquired from the original CT data, 15.63±8.06 mm for the STL files, and 14.47±7.71 mm for the 3D printed liver model. The 3D liver model did not enhance the radiologists' perception of patient-specific anatomy or pathology. Kappa analysis of the surgeon's responses to survey questions yielded a percentage agreement of 80%, and a κ value of 0.38 (P=0.24) indicating fair agreement. | 4 |
| 13 | Kuroda S, Kobayashi T, Ohdan H. 3D printing model of the intrahepatic vessels for navigation during anatomical resection of hepatocellular carcinoma. Int J Surg Case Rep. 2017;41:219–222. | Review/Other-Tx | 2 | Authors report the use of the model for anatomical resection of hepatocellular carcinoma. | Two simplified 3D models of intrahepatic vessels without liver parenchyma were developed and used in surgical planning. Models were found to be helpful in guiding hepatic resections. | 4 |
| 14 | Witowski JS, Pędziwiatr M, Major P, Budzyński A. Cost-effective, personalized, 3D-printed liver model for preoperative planning before laparoscopic liver hemihepatectomy for colorectal cancer metastases. Int J Comput Assist Radiol Surg. 2017;12(12):2047–2054. | Review/Other-Tx | 1 | This paper presents a cost-effective technique of preparing 3D-printed liver models that preserves the shape and all of the structures, including the vessels and the tumor, which in the present case is colorectal liver metastasis. | As a result, a transparent, full-sized liver model with visible vessels and colorectal metastasis was created for under $150, which-taking into account 3D printer prices-is much cheaper than models presented in previous research papers. | 4 |
| 15 | Yang Y, Zhou Z, Liu R, Chen L, Xiang H, Chen N. Application of 3D visualization and 3D printing technology on ERCP for patients with hilar cholangiocarcinoma. Exp Ther Med. 2018;15(4):3259–3264. | Observational-Dx and Tx | 15 | The aim of the present study as to evaluate the clinical value of three-dimensional visualization (3DV) and 3D printing (3DP) technologies for ERCP in patients with hepatocellular carcinoma. | 3DV and 3DP models were successfully constructed for all patients, which presented the tumor, bile duct and the spatial relationship between them from multiple perspectives. The ERCP of all patients in the present study were performed successfully. The diagnostic accuracy of BC type results by 3DV and 3DP models was 93.3%. | 3 |
| 16 | Choi YR, Kim JH, Park SJ, Hur BY, Han JK. Therapeutic response assessment using 3D ultrasound for hepatic metastasis from colorectal cancer: Application of a personalized, 3D-printed tumor model using CT images. PLoS ONE. 2017;12(8):e0182596. | Observational-Dx and Tx | 20 | To evaluate accuracy and reliability of three-dimensional ultrasound (3D US) for response evaluation of hepatic metastasis from colorectal cancer (CRC) using a personalized 3D-printed tumor model. | 3D US tumor volume showed no significant difference from CT volume (7.18 ± 5.44 mL, 8.31 ± 6.32 mL vs 7.42 ± 5.76 mL in CT, p>0.05). 3D US provided a high correlation coefficient with CT (r = 0.953, r = 0.97) as well as a high inter-observer intraclass correlation (0.978; 0.958-0.988). Regarding response, 3D US was in agreement with CT in 17 and 18 out of 20 patients for observer 1 and 2 with excellent agreement (κ = 0.961). There was no significant difference between 3D printed model and reference volume from CT images. | 3 |
| 17 | Trout AT, Batie MR, Gupta A, Sheridan RM, Tiao GM, Towbin AJ. 3D printed pathological sectioning boxes to facilitate radiological-pathological correlation in hepatectomy cases. J Clin Pathol. 2017;70(11):984–987. | Observational-Dx | 13 | The purpose of this work is to present a technique to standardize hepatic sectioning by creating 3D printed specimen boxes based on CT and MRI. | Authors used 3D printed specimen boxes to facilitate pathological sectioning and observed close correlation between imaging and gross pathology in patients with both unifocal and multifocal tumors.. | 3 |
| 18 | Yang T, Tan T, Yang J, et al. The impact of using three-dimensional printed liver models for patient education. J Int Med Res. 2018;46(4):1570–1578. | Observational-Tx | 7 | To investigate the impact of using a three-dimensional (3D) printed liver model for patient education. | Seven children and their 14 parents were enrolled in the study. After the presentation of 3D printed models, parental understanding of basic liver anatomy and physiology, tumour characteristics, the planned surgical procedure, and surgical risks significantly improved. Parents demonstrated improvements in their understanding of basic liver anatomy by 26.4%, basic liver physiology by 23.6%, tumour characteristics by 21.4%, the planned surgical procedure by 31.4%, and surgical risks by 27.9%. | 3 |
| 19 | Zeng N, Fang CH, Fan YF, et al. [The construction of three-dimensional visualization platform and its application in diagnosis and treatment for hilar cholangiocarcinoma]. Zhonghua Wai Ke Za Zhi. 2016;54(9):680–685. | Observational-Dx and Tx | 32 | To explore the construction and clinical efficacy of three-dimensional(3D) visualization platform about diagnosis and treatment of hilar cholangiocarcinoma. | Developed 3D models clearly showed intrahepatic anatomy, size and location of tumors, relationship between tumor and intrahepatic vasculature. | 2 |
| 20 | Fang C, Fang Z, Fan Y, Li J, Xiang F, Tao H. [Application of 3D visualization, 3D printing and 3D laparoscopy in the diagnosis and surgical treatment of hepatic tumors]. Nan Fang Yi Ke Da Xue Xue Bao. 2015;35(5):639–645. | Observational-Dx and Tx | 22 | To study the value of three-dimensional (3D) visualization, 3D printing and 3D laparoscopy (3-3D techniques) in the diagnosis and surgical treatment of hepatic tumors. | 3D printed models were created and used for surgical simulation before procedure. Authors claim that models clearly displayed tumor location. | 2 |
| 21 | Zein NN, Hanouneh IA, Bishop PD, et al. Three-dimensional print of a liver for preoperative planning in living donor liver transplantation. Liver Transpl. 2013;19(12):1304–1310. | Observational-Tx | 6 | To establish anatomical precision and volumetric accuracy in 3D printed models for donors and recipients undergoing LDLT. | Using standardized preoperative, intraoperative, and postoperative assessments, authors demonstrated identical anatomical and geometrical landmarks in the 3D-printed models and native livers. | 3 |
| 22 | Hu M, Hu H, Cai W, et al. The Safety and Feasibility of Three-Dimensional Visualization Technology Assisted Right Posterior Lobe Allied with Part of V and VIII Sectionectomy for Right Hepatic Malignancy Therapy. J Laparoendosc Adv Surg Tech A. 2018;28(5):586–594. | Observational-Dx and Tx | 9 | 3D printed models were used to provide intraoperative guidance in patients undergoing right posterior lobe allied with part of V and VIII sectionectomy. | Printed model were used to identify middle and right hepatic veins and determine resection line. | 2 |
| 23 | Wang J-Z, Xiong N-Y, Zhao L-Z, Hu J-T, Kong D-C, Yuan J-Y. Review fantastic medical implications of 3D-printing in liver surgeries, liver regeneration, liver transplantation and drug hepatotoxicity testing: A review. Int J Surg. 2018;56:1–6. | Review/Other-Tx | NA | Authors comment on applications of 3D printing and 3D bioprinting, including patient-specific liver models for medical education and surgical simulations. | No results stated in abstract. | 4 |
| 24 | Oshiro Y, Ohkohchi N. Three-Dimensional Liver Surgery Simulation: Computer-Assisted Surgical Planning with Three-Dimensional Simulation Software and Three-Dimensional Printing. Tissue Eng Part A. 2017;23(11–12):474–480. | Review/Other-Tx | NA | Herein, authors review the history of virtual hepatectomy using computer-assisted surgery (CAS) and our research to date, and we discuss the future prospects of CAS. | Authors developed a novel hollow 3D-printed liver model whose surface is covered with frames. This model was found useful for safe liver resection, has better visibility, and the production cost was reduced to one-third of a previously reported models. | 4 |
| 25 | Yao R, Xu G, Mao S-S, et al. Three-dimensional printing: review of application in medicine and hepatic surgery. Cancer Biol Med. 2016;13(4):443–451. | Review/Other-Tx | NA | Three-dimensional (3D) printing (3DP) is a rapid prototyping technology that has gained increasing recognition in many different fields. Inherent accuracy and low-cost property enable applicability of 3DP in many areas, such as manufacturing, aerospace, medical, and industrial design. | No results stated in abstract. | 4 |
| 26 | Alkhouri N, Zein NN. Three-dimensional printing and pediatric liver disease. Curr Opin Pediatr. 2016;28(5):626–630. | Review/Other-Tx | NA | The purpose of the review is to provide a concise summary of the role of three-dimensional printing technology as it relates to the field of pediatric hepatology and liver transplantation | Authors demonstrated the feasibility of printing three-dimensional livers with identical anatomical and geometrical landmarks to the native liver to facilitate presurgical planning of complex liver surgeries. Medical educators are exploring the use of three-dimensional printed organs in anatomy classes and surgical residencies. Moreover, mini-livers are being developed by regenerative medicine scientist as a way to test new drugs and, eventually, whole livers will be grown in the laboratory to replace organs with end-stage disease solving the organ shortage problem. | 4 |
| 27 | Widmann G, Wallach D, Toporek G, Schullian P, Weber S, Bale R. Angiographic C-arm CT- versus MDCT-guided stereotactic punctures of liver lesions: nonrigid phantom study. AJR Am J Roentgenol. 2013;201(5):1136–1140. | Review/Other-Tx | NA | Our purpose was to evaluate the accuracy of using C-arm CT for single and multimodality image fusions and to compare the targeting accuracy of liver lesions with the reference standard of MDCT. | Target registration error of the image fusion showed no significant difference (p > 0.05) between both modalities. In five series with a total of 25 punctures for each modality, the lateral target positioning error (i.e., the lateral distance between the needle tip and the planned trajectory) was similar for C-arm CT (mean [± SD], 1.6 ± 0.6 mm) and MDCT (1.82 ± .97 mm) (p = 0.33). | 4 |
| 28 | Witowski J, Wake N, Grochowska A, et al. Investigating accuracy of 3D printed liver models with computed tomography. Quant Imaging Med Surg. 2019;9(1):43-52. | Observational-Dx and Tx | 15 | The aim of this study was to evaluate the accuracy of three-dimensional (3D) printed liver models developed by a cost-effective approach for establishing validity of using these models in a clinical setting. | The median liver volume in MCT was 1,281.84 [interquartile range (IQR) =296.86] cm3, and 1,448.03 (IQR =413.23) cm3 in PCT. Analysis of differences between surfaces showed that the median value of mean Hausdorff distances for liver parenchyma was 1.92 mm. Bland-Altman plots revealed no significant bias in liver volume and diameters of hepatic veins and tumor location. Median errors of all measured vessel diameters were smaller than CT slice height. There was a slight trend towards undersizing anatomical structures, although those errors are most likely due to source imaging. | 2 |
| 29 | Tang R, Ma L, Li A, et al. Choledochoscopic Examination of a 3-Dimensional Printing Model Using Augmented Reality Techniques: A Preliminary Proof of Concept Study. Surg Innov. 2018;25(5):492–498. | Review/Other-Tx | NA | Investigated use of augmented reality for additional guidance during choledochoscopic examination using 3D printed model of the patient’s biliary tree. | Training choledochoscopy was performed on the 3D printed model. The choledochoscope was guided into the left and right hepatic ducts, the right anterior hepatic duct, the bile ducts of segment 8, the hepatic duct in subsegment 8, the right posterior hepatic duct, and the left and the right bile ducts of the caudate lobe. Although stability in tracking was less than ideal, the virtual choledochoscope images and EM sensor tracking were effective for navigation. | 4 |
| 30 | Holt BA, Hearn G, Hawes R, Tharian B, Varadarajulu S. Development and evaluation of a 3D printed endoscopic ampullectomy training model (with video). Gastrointest Endosc. 2015;81(6):1470-1475.e5. | Observational-Tx | NA | To develop a training model that can be used to improve technical skills, knowledge, and confidence in performing endoscopic ampullectomy. | Endoscopists performed an ampullectomy on 3D printed model. The mean overall technical and visual realism scores were 3.1 (standard deviation [SD], 0.9) and 3.2 (SD, 0.9), respectively. Ten participants (10/15, 66.7%) thought that their technical knowledge had improved, and 11 thought that it would increase further with additional sessions (11/15, 73.3%). Mean confidence score before and after using the model was 2.2 (SD, 1.2) and 2.9 (SD, 1.1), respectively (P=.132). | 3 |
| 31 | Dhir V, Itoi T, Fockens P, et al. Novel ex vivo model for hands-on teaching of and training in EUS-guided biliary drainage: creation of “Mumbai EUS” stereolithography/3D printing bile duct prototype (with videos). Gastrointest Endosc. 2015;81(2):440–446. | Observational-Tx | NA | To evaluate a stereolithography/3-dimensional (3D) printing bile duct prototype for teaching and training in EUS-guided biliary drainage (EUS-BD). | Fifteen participants returned the questionnaire, and 10 completed all 4 steps of EUS-BD. The median score for overall utility was 4, whereas that for EUS and US views was 5. Participants with experience in performing more than 20 EUS-BD procedures scored the prototype significantly lower for stent placement (P = .013) and equivalent for needle puncture, tract dilation, and wire manipulation. The success rate of various steps was 100% for needle puncture and tract dilation, 82.35% for wire manipulation, and 80% for stent placement. The mean overall procedure time was 18 minutes. | 3 |
| 32 | Study Group of Pancreatic Surgery in Chinese Society of Surgery of Chinese Medical Association, Pancreatic Committee of Chinese Research Hospital Association, Digital Medicine Branch of Chinese Medical Association, Digital Medicine Committee of Chinese Research Hospital Association. [Expert consensus of precise diagnosis and treatment for pancreatic head cancer using three-dimensional visualization technology]. Zhonghua Wai Ke Za Zhi. 2017;55(12):881–886. | Review/Other-Tx | NA | In order to standardize the application of 3D visualization and 3D printing technology in the diagnosis and treatment of pancreatic head cancer, Chinese experts in relevant fields were organized by four committees to formulate this expert consensus. | No results stated in abstract. | 4 |
| 33 | Yang YY, Huang HG. [Development status of three-dimensional printing technology in pancreatic surgery]. Zhonghua Wai Ke Za Zhi. 2017;55(10):795–797. | Review/Other-Tx | NA | This article is a summary of three-dimensional printing technology in pancreatic surgery, in order to recognize the developmental level and research progress of three-dimensional printing technology, and to give advices about the research prospect and development direction in pancreatic surgery field. | No results stated in abstract. | 4 |
| 34 | Marconi S, Pugliese L, Botti M, et al. Value of 3D printing for the comprehension of surgical anatomy. Surg Endosc. 2017;31(10):4102–4110. | Observational-Tx | 15 | To assess whether 3D printed model could be more informative than standard MDCT images and digital 3D visualizations in predicting the real anatomy of the case. | The visual and tactile inspection of 3D models allowed the best anatomical understanding, with faster and clearer comprehension of the surgical anatomy. As expected, less experienced medical students perceived the highest benefit (53.9% ± 4.14 of correct answers with 3D-printed models, compared to 53.4 % ± 4.6 with virtual models and 45.5% ± 4.6 with MDCT), followed by surgeons and radiologists. The average time spent by participants in 3D model assessing was shorter (60.67 ± 25.5 s) than the one of the corresponding virtual 3D reconstruction (70.8 ± 28.18 s) or conventional MDCT scan (127.04 ± 35.91 s). | 3 |
| 35 | Sampogna G, Pugliese R, Elli M, Vanzulli A, Forgione A. Routine clinical application of virtual reality in abdominal surgery. Minim Invasive Ther Allied Technol. 2017;26(3):135–143. | Observational-Tx | 15 | Authors investigate feasibility, user appreciation and clinical impact of 3D reconstruction, virtual reality and 3D printing in abdominal surgery. | Preoperative surgical planning and intraoperative guidance was feasible for all patients included in the study. The vast majority of surgeons interviewed scored their quality and usefulness as very good. | 3 |
| 36 | Andolfi C, Plana A, Kania P, Banerjee PP, Small S. Usefulness of Three-Dimensional Modeling in Surgical Planning, Resident Training, and Patient Education. J Laparoendosc Adv Surg Tech A. 2017;27(5):512–515. | Review/Other-Tx | 1 | In this paper, authors developed a 3D digital model of a cancer of the head of the pancreas by integrating actual CT data with 3D modeling process. After this process, the virtual pancreatic model was also produced using a high-quality 3D printer. | Reconstructing and compositing the different series together enhanced the imaging, which allowed clearer observations of the relationship between the mass and the blood vessels, and evidence that the tumor was unresectable. Data files were converted for printing a 100% size rendering model, used for didactic purposes and to discuss with the patient. | 4 |
| 37 | Marconi S, Pugliese L, Del Chiaro M, Pozzi Mucelli R, Auricchio F, Pietrabissa A. An innovative strategy for the identification and 3D reconstruction of pancreatic cancer from CT images. Updates Surg. 2016;68(3):273–278. | Observational-Dx | 10 | To propose semi-automatic workflow for pancreatic ductal adenocarcinoma 3D reconstruction and 3D printing from MDCT. | In seven of ten cases, the 3D reconstruction is accepted without any modification, while in three cases, only 1.88, 5.13, and 5.70 %, respectively, of the segmentation labels are modified, preliminary proving the high effectiveness of the tool. | 3 |
| 38 | Zheng Y, Yu D, Zhao J, Wu Y, Zheng B. 3D Printout Models vs. 3D-Rendered Images: Which Is Better for Preoperative Planning? J Surg Educ. 2016;73(3):518–523. | Review/Other-Tx | NA | In this study, we presented 3 different pancreatic cancer cases to surgical residents in the form of 3D-rendered images and 3D-printed models to investigate which modality resulted in the most appropriate preoperative plan. | Residents in group B showed significantly higher quality of the surgical plan scores compared with residents in group A (76.4 ± 10.5 vs. 66.5 ± 11.2, p = 0.018). This difference was due in large part to a significant difference in knowledge of key surgical steps (22.1 ± 2.9 vs. 17.4 ± 4.2, p = 0.004) between each group. All participants reported a high level of satisfaction with the exercise. | 3 |
| 39 | Mahmoud A, Bennett M. Introducing 3-Dimensional Printing of a Human Anatomic Pathology Specimen: Potential Benefits for Undergraduate and Postgraduate Education and Anatomic Pathology Practice. Arch Pathol Lab Med. 2015;139(8):1048–1051. | Review/Other-Dx and Tx | NA | To apply 3D printing in anatomic pathology for teaching, training, and clinical correlation purposes. | Three-dimensionally printed models of anatomic pathology specimens created included pancreatoduodenectomy (Whipple operation) and radical nephrectomy specimens. The models accurately depicted the topographic anatomy of selected specimens and illustrated the anatomic relation of excised lesions to adjacent normal tissues. | 4 |
| 40 | Pietrabissa A, Marconi S, Peri A, et al. From CT scanning to 3-D printing technology for the preoperative planning in laparoscopic splenectomy. Surg Endosc. 2016;30(1):366–371. | Observational-Tx | 12 | To assess the value of patient-specific 3-D physical manufacturing of spleno-pancreatic anatomy in helping during patient's counseling and for preoperative planning. | The post-processing analysis required an average of 2; 20 h was needed to physically print each model and 4 additional hours to finalize each object. The cost for the material employed for each object was around 300 euros. Ten patients gave a score of 5, two a score of 4. Six residents gave a score of 5, four a score of 4. | 4 |
| 41 | Williams A, McWilliam M, Ahlin J, Davidson J, Quantz MA, Bütter A. A simulated training model for laparoscopic pyloromyotomy: Is 3D printing the way of the future? J Pediatr Surg. 2018;53(5):937–941. | Observational-Tx | NA | The purpose of this study was to validate a 3D HPS stomach model and assess model reliability and surgical realism. | Participants reported their experience levels as novice (22%), inexperienced (26%), intermediate (19%), and experienced (33%). Interrater reliability was similar for overall average GOALS and Task Specific Assessments (TSA) scores. There was a significant improvement in GOALS (p<0.0001) and TSA scores (p=0.03) between attempts and overall. Participants felt the model accurately simulated a laparoscopic pyloromyotomy (82%) and would be a useful tool for beginners (100%). | 3 |
| 42 | Lee S, Ahn JY, Han M, et al. Efficacy of a Three-Dimensional-Printed Training Simulator for Endoscopic Biopsy in the Stomach. Gut Liver. 2018;12(2):149–157. | Observational-Tx | NA | Authors used 3D printing to create a new biopsy simulator for the stomach and investigated its efficacy and realism in endoscopic biopsy training. | The faculty members strongly agreed that the simulator realistically reflected endoscopic handling and was reasonable for endoscopic training (scores of 6.2±0.8 and 6.4±0.9, respectively). Importantly, experienced endoscopists reported that the difficulty levels of the 10 biopsy sites in the simulator were a realistic match for the actual stomach. | 3 |
| 43 | Kim GB, Park J-H, Song H-Y, et al. 3D-printed phantom study for investigating stent abutment during gastroduodenal stent placement for gastric outlet obstruction. 3D Print Med. 2017;3(1):10. | Review/Other-Tx | NA | An in vitro experiment using a flexible anthropomorphic three-dimensional (3D)-printed phantom of gastroduodenal strictures for simulation of placing a self-expandable metallic stent. | Flexible antrophomorphic 3D printed gastroduodenal phantom study revealed that stent abutment can cause prolonged passage of soft and solid diets through the stent. | 4 |
| 44 | Garcia-Granero A, Sánchez-Guillén L, Fletcher-Sanfeliu D, et al. Application of three-dimensional printing in laparoscopic dissection to facilitate D3-lymphadenectomy for right colon cancer. Tech Coloproctol. 2018;22(2):129–133. | Review/Other-Tx | 1 | To use 3D printing in planning laparoscopic dissection of the gastrocolic trunk of Henle and surgical trunk of Gillot during D3-lymphadenectomy. | Printed model was used to improve planning of laparoscopic right hemicolectomy for right colon cancer and aided decision making concerning the extent of surgery. | 4 |
| 45 | Luzon JA, Andersen BT, Stimec BV, et al. Implementation of 3D printed superior mesenteric vascular models for surgical planning and/or navigation in right colectomy with extended D3 mesenterectomy: comparison of virtual and physical models to the anatomy found at surgery. Surg Endosc. 2018; | Observational-Tx | 22 | The objective of this study was to evaluate the linear dimensional anatomy landmark differences of the superior mesenteric artery and vein between (1) 3D virtual models, (2) 3D printouts, and (3) peroperative measurements. | Four parameters were measured: distance between the origins of the ileocolic and the middle colic artery, distance between the termination of the gastrocolic trunk and the ileocolic vein, and the calibers of the middle colic and ileocolic arteries. The inter-arterial distance has proven a strong correlation between all the three modalities implied (Pearson's coefficient 0.968, 0.956, 0.779, respectively), while inter-venous distances showed a weak correlation between peroperative measurements and both virtual and physical models. | 3 |
| 46 | Hamabe A, Ito M. A three-dimensional pelvic model made with a three-dimensional printer: applications for laparoscopic surgery to treat rectal cancer. Tech Coloproctol. 2017;21(5):383–387. | Review/Other-Dx and Tx | 2 | To help understand the three-dimensional (3D) spatial relationships among the highly complex structures of the pelvis, we made a novel 3D pelvic model with a 3D printing system. | The models clearly demonstrated the complicated spatial relationships between anatomical structures in the pelvis. | 4 |
| 47 | Tominaga T, Takagi K, Takeshita H, et al. Usefulness Of Three-Dimensional Printing Models for Patients with Stoma Construction. Case Rep Gastroenterol. 2016;10(1):57–62. | Observational-Tx | 5 | The aim of this study was to clarify whether the use of patient-specific stoma models is helpful for patient education. | All patients were educated about their stoma and potential stoma-associated problems using three-dimensional stoma models, and all practiced cutting face plates using three-dimensional face plates. The models were also used during medical staff conferences to discuss current issues. All patients understood their problems and finally became self-reliant. | 3 |
| 48 | Sahnan K, Adegbola SO, Tozer PJ, et al. Innovation in the imaging perianal fistula: a step towards personalised medicine. Therap Adv Gastroenterol. 2018;11:1756284818775060. | Review/Other-Dx | 3 | To explore alternative platforms better to understand complex perianal fistulas through three-dimensional (3D) imaging and reconstruction. | Three examples of 3D printed models demonstrating complex perianal fistula were created. The anatomical components are displayed in different colours: red: fistula tract; green: external anal sphincter and levator plate; blue: internal anal sphincter and rectum. One of the models was created to be split in half, to display the internal opening and allow complexity in the intersphincteric space to better evaluated. | 4 |
| 49 | Nishihara Y, Isobe Y, Kitagawa Y. Validation of newly developed physical laparoscopy simulator in transabdominal preperitoneal (TAPP) inguinal hernia repair. Surg Endosc. 2017;31(12):5429–5435. | Review/Other-Tx | NA | The purpose of this study was to create a novel physical simulator for TAPP inguinal hernia repair and obtain surgeons' opinions regarding its efficacy. | All participants strongly agreed that the 3D-printed physical simulator and organ replica model were highly useful for TAPP inguinal hernia repair training (median, 5 points) and TAPP inguinal hernia repair education (median, 5 points). They felt that the simulator would be effective for TAPP inguinal hernia repair training before entering the operating theater. All surgeons considered that this simulator should be introduced in the residency curriculum. | 4 |
| **Supplementary Table**. Grading of each included study with a strength of evidence assessment according to ACR Appropriateness Criteria Evidence Document.^50^ Studies were categorized as either primarily diagnostic (Dx), therapeutic (Tx), or both (Dx and Tx) along with a designation of observational, experimental, or review/other category. The review/other category is designated for studies that did not meet the definitions the ACR Evidence Document^50^ for observational or experimental studies. | | | | | | |
